# Supplementary material for: Modular deregulation of central carbon metabolism for efficient xylose utilization in Saccharomyces cerevisiae
Source: Nat Commun. 2025 May 16;16:4551. doi: 10.1038/s41467-025-59966-x (PMC12084563; doi:10.1038/s41467-025-59966-x)
Supplement: Supplementary file 1 — Supplementary information [file 41467_2025_59966_MOESM1_ESM.pdf]

Supplementary Information

for

Modular deregulation of central carbon metabolism for efficient xylose utilization in Saccharomyces cerevisiae

Xiaowei Li, Yanyan Wang, Xin Chen, Leon Eisentraut, Chunjun Zhan, Jens Nielsen, and Yun Chen

Supplementary Figures

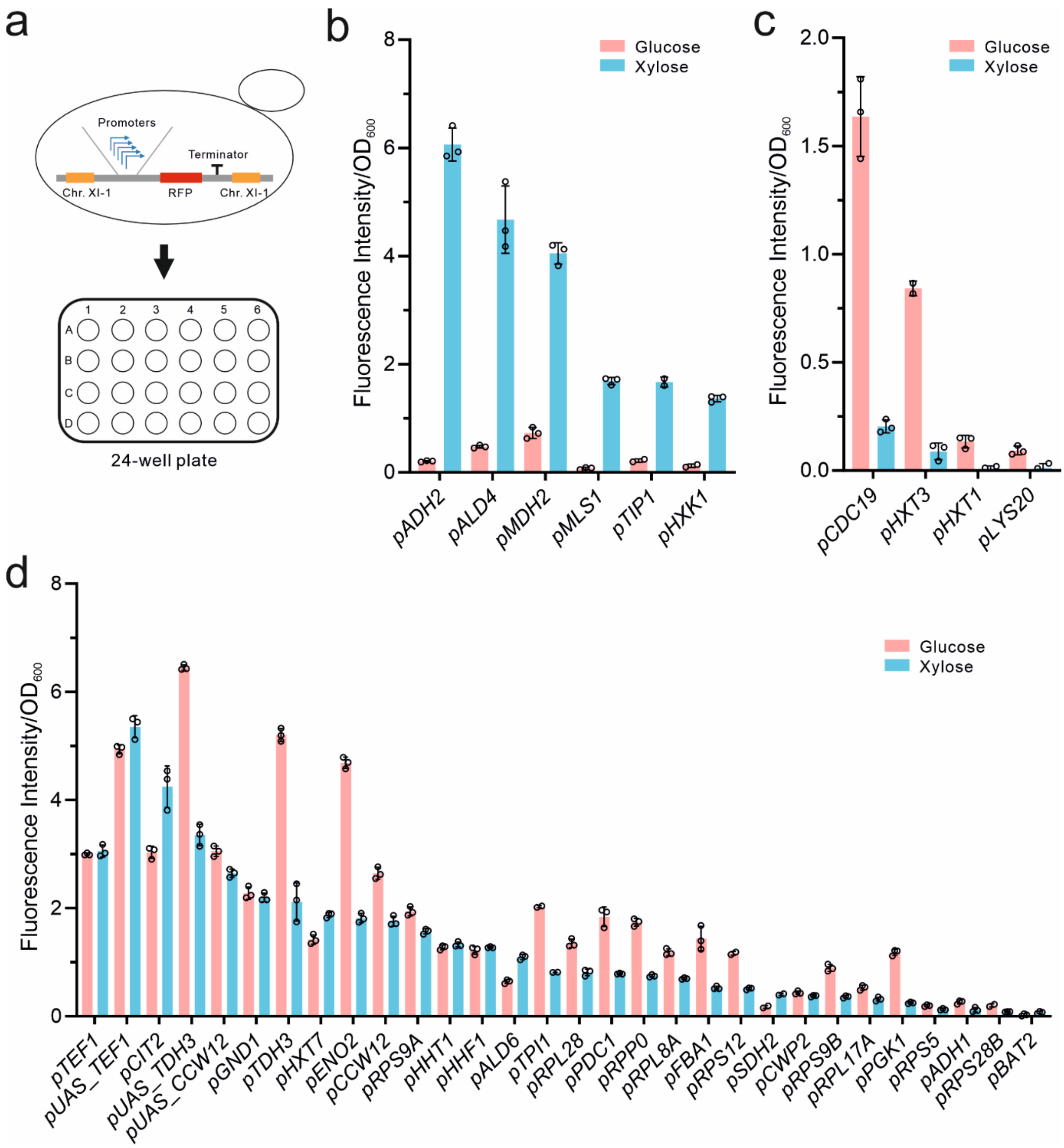

Supplementary Fig. 1 Evaluating various promoters with glucose or xylose.

a, Schematic depiction of the promoter testing procedure. The promoter under investigation was integrated at the yeast chromosome XI-1 locus. Red fluorescent protein (RFP) served as a reporter. The derived strains were cultured in a 24-well flower plate using the BioLector, with either 2% glucose or xylose as the carbon source. **b-d**, Classification of tested promoters. Promoters were categorized into three groups: xylose-responsive promoters (**b**); glucose-responsive promoters (**c**); and constitutive promoters (**d**). The promoter *pTEF1* was used as internal standard. All data presented are the average from n=3 biologically independent samples, with error bars representing the standard deviation. Source data are provided as a Source Data file.

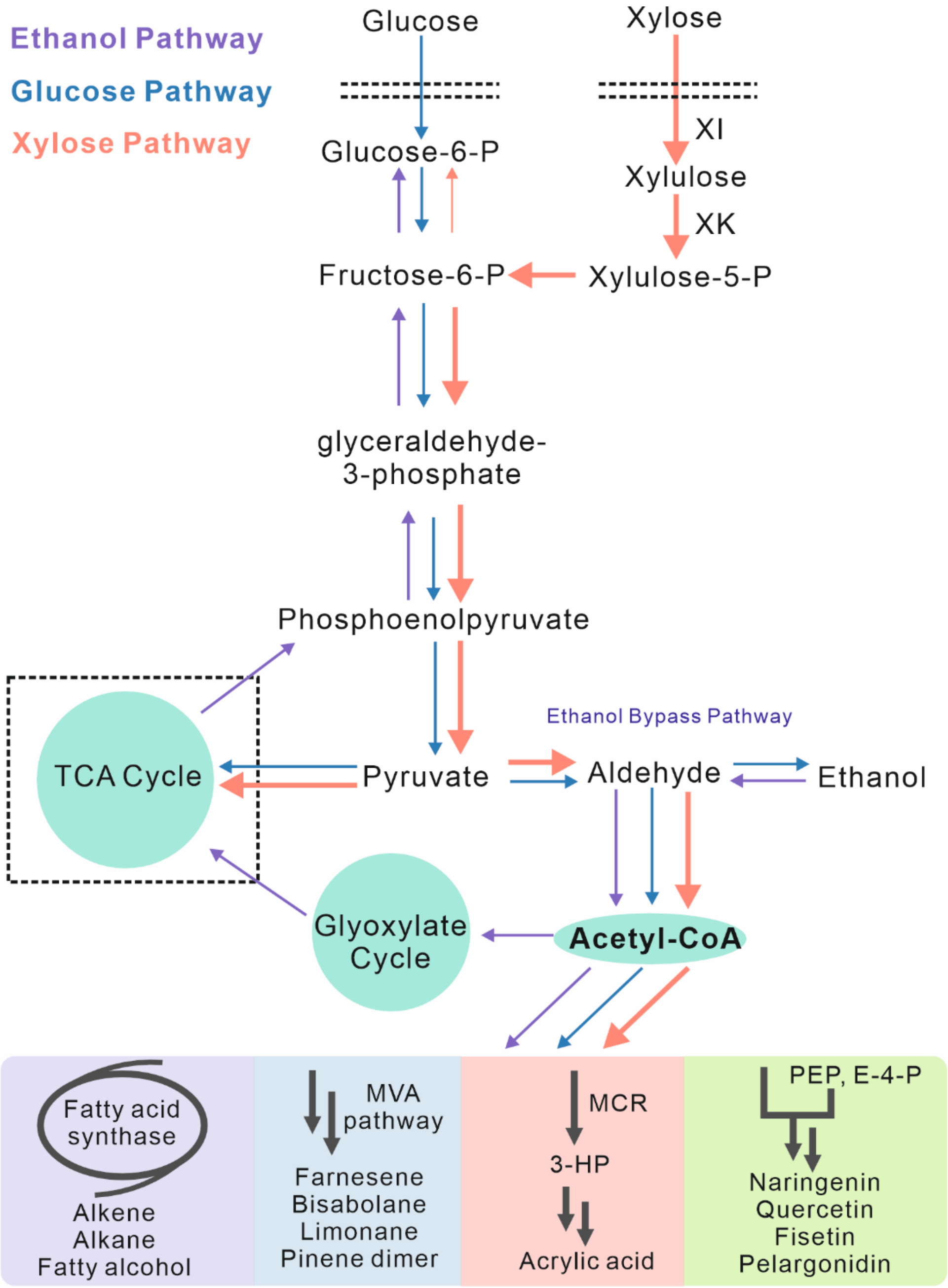

Supplementary Fig. 2 Metabolic pathways for carbon sources originating from ethanol, glucose, or xylose.

When cells grow on glucose, they exhibit strong glycolysis, whereas a robust respiratory state is observed during growth on ethanol. For xylose metabolism, our understanding of intracellular regulation of central carbon remains limited. From a metabolic pathway perspective, xylose metabolism should be better aligned with glucose metabolism to generate acetyl-CoA, as both xylose and glucose are processed in a top-down manner and have the overflow metabolism for acetyl-CoA formation. Among these carbon sources, acetyl-CoA stands out as a crucial intermediate, bridging central carbon metabolism with the formation of products such as fatty acid derivatives, terpenoids, 3-HP, and flavonoids. Notably, the production of 3-HP is streamlined, requiring only one enzyme, which bypasses the need for certain intermediate metabolites. The arrows for xylose metabolism and biochemical generation are highlighted in bold.

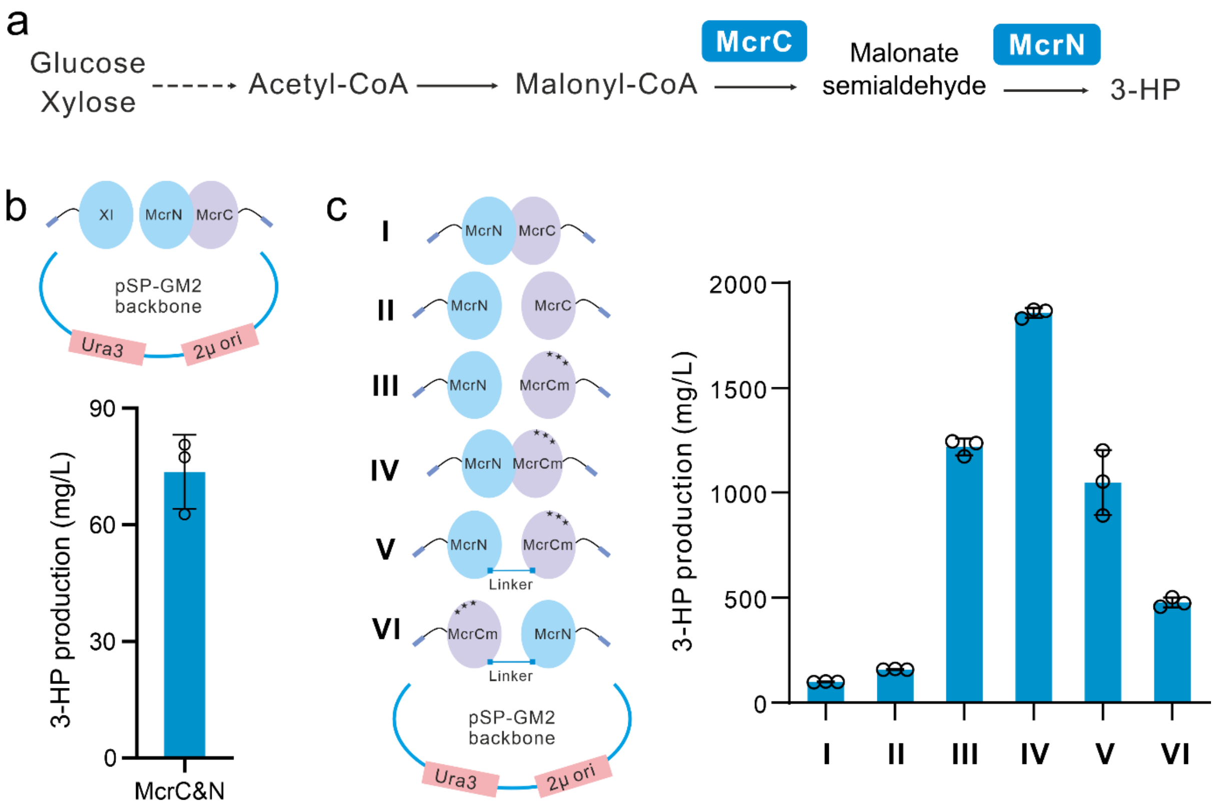

Supplementary Fig. 3 Optimizing the expression of MCR in plasmids.

a, Schematic representation of 3-HP synthesis from malonyl-CoA. The MCR protein consists of two distinct segments: McrN, responsible for the second step, and McrC, handling the first step. Malonate semialdehyde acts as the intermediate in this process. **b**, 3-HP production using MCR overexpressing plasmid. Both the xylose isomerase (XI) enzyme and MCR (McrN/McrC) were incorporated into a high-copy plasmid. Strains containing this plasmid were cultivated in a minimal medium with xylose as the sole carbon source. **c**, 3-HP Production from glucose. To streamline the setup and facilitate comparison between two distinct carbon sources, MCR was incorporated into a plasmid with glucose as the chosen carbon source. I: The wild-type MCR (McrN/McrC) was driven by the *pTEF1* promoter. II: MCR was divided into McrN and McrC, regulated by *pPGK1* and *pTEF1* promoters, respectively. III: Three specific mutations (N940V, K1106W, S1114R) were introduced into the separated McrC, producing McrCm. IV: The same three mutations were added to the McrC portion of the original MCR. V: McrN was fused to McrCm using the GSTSSGSG linker. VI: McrCm was joined with McrN using the GSTSSGSG linker. For constructs IV-VI, the *pTEF1* promoter directed gene expression. All data presented are the average from n=3 biologically independent samples, with error bars representing the standard deviation. Source data are provided as a Source Data file.

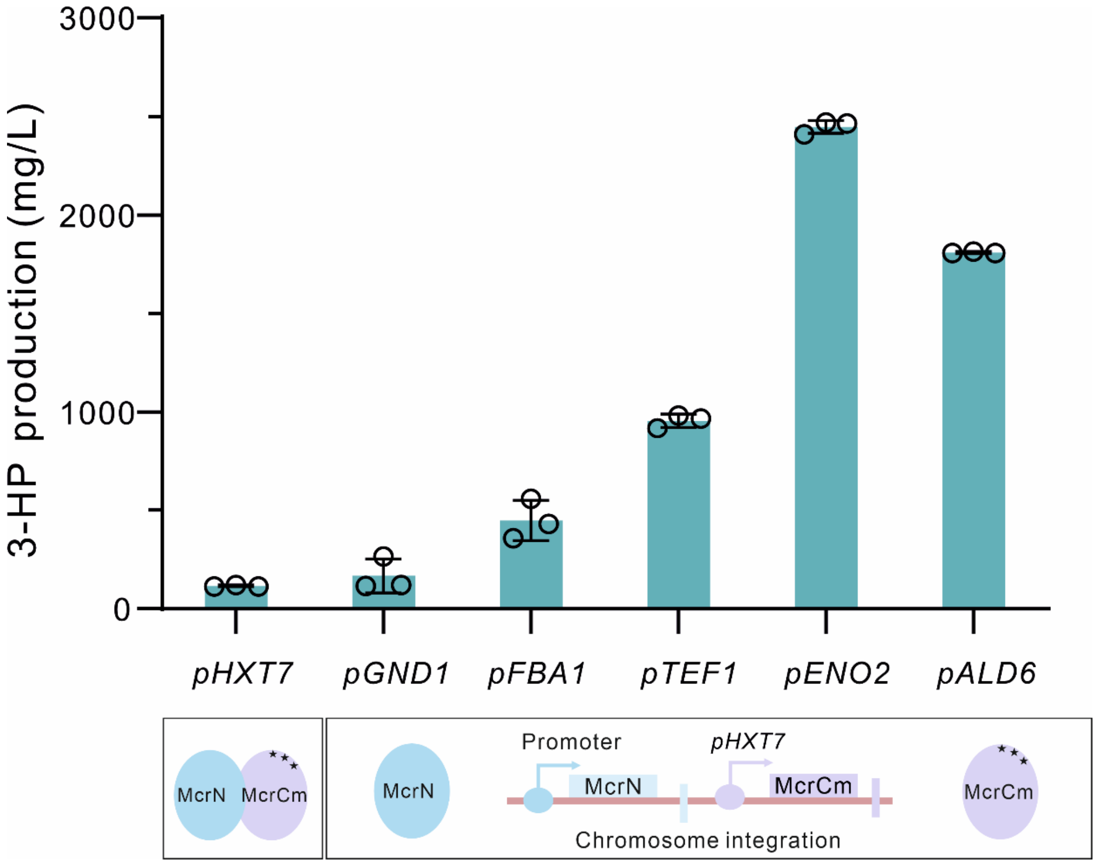

Supplementary Fig. 4 Optimizing the expression of MCR in genome.

McrN and McrCm were incorporated into the yeast chromosome at the XI-3 locus. While the *pTEF1* promoter drove McrCm, 6 different constitutive promoters were employed to regulate McrN expression. Strains were grown in a minimal medium with 2% glucose as the carbon source. All data presented are the average from n=3 biologically independent samples, with error bars representing the standard deviation. Source data are provided as a Source Data file.

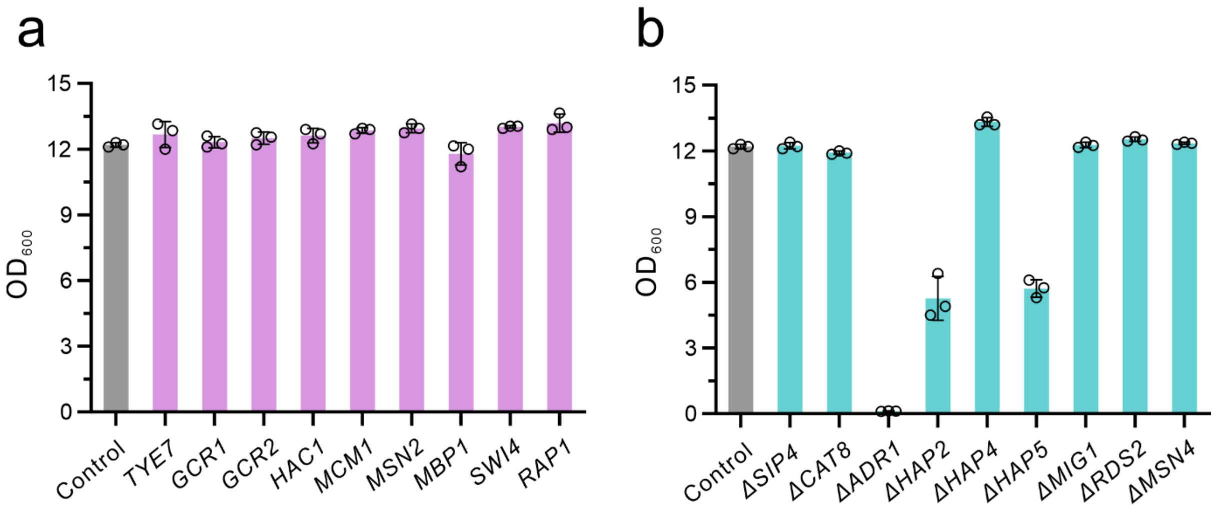

Supplementary Fig. 5 Growth of TFs engineered strains.

a, Final OD values for strains overexpressing TFs. **b**, Final OD values for strains with TFs deletion. Strain R30c was used as the control. All strains were cultivated in a defined minimal medium with 2% xylose as the sole carbon source. All data represent the mean derived from n=3 biologically independent samples, with error bars indicating the standard deviation. Source data are provided as a Source Data file.

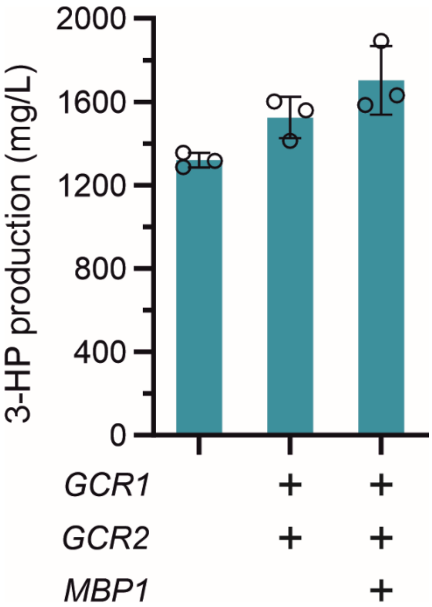

Supplementary Fig. 6 Conditional expression of TFs to increase 3-HP production.

Three TFs (*GCR1*, *GCR2*, and *MBP1*) were conditionally expressed in strain R30c. Strains were grown in a defined minimal medium with 2% xylose as the sole carbon source. All data represent the mean derived from n=3 biologically independent samples, with error bars indicating the standard deviation. Source data are provided as a Source Data file.

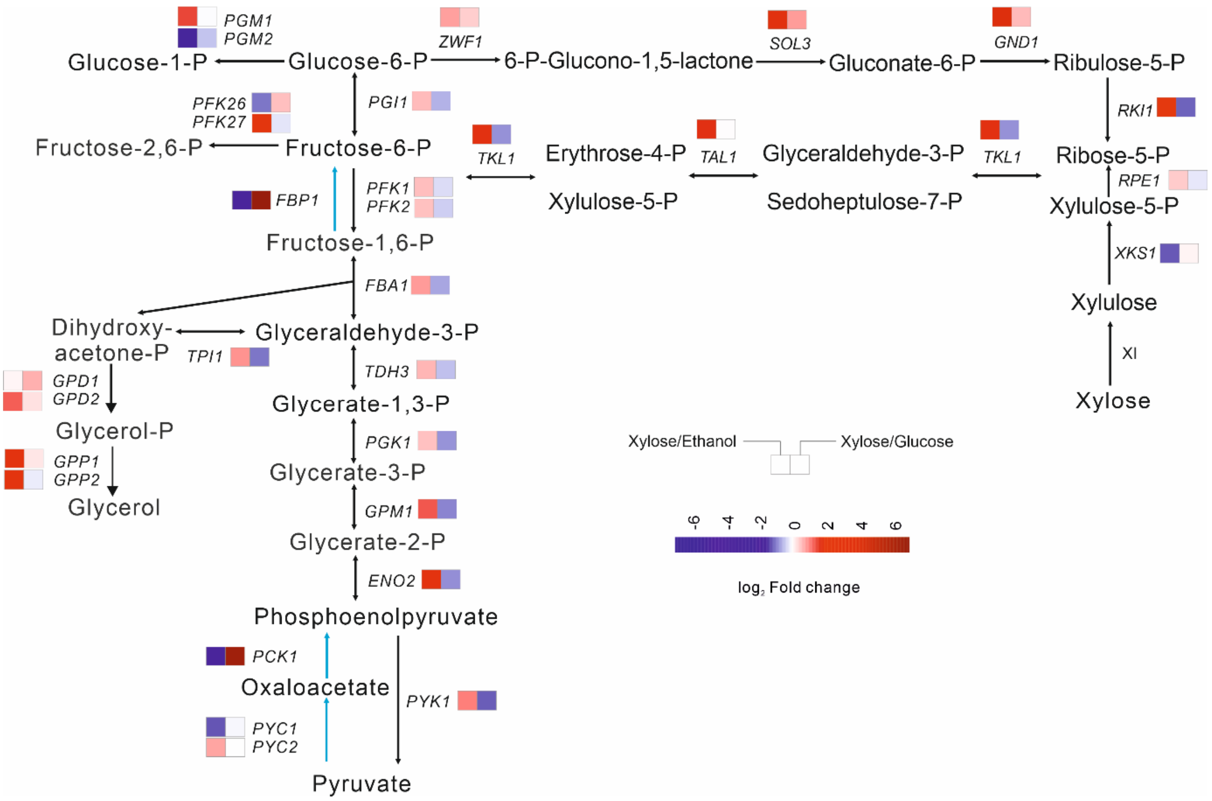

Supplementary Fig. 7 Transcriptional changes of genes involved in glycolysis, gluconeogenesis, and PP pathway.

The left squares represent transcriptional fold changes for strains grown on xylose versus ethanol, while the right squares represent transcriptional fold changes for strains grown on xylose versus glucose. Black arrows show glycolysis and the PP pathway, while cyan arrows indicate gluconeogenesis. Genes for each catalytic step are labeled adjacent to the arrows.

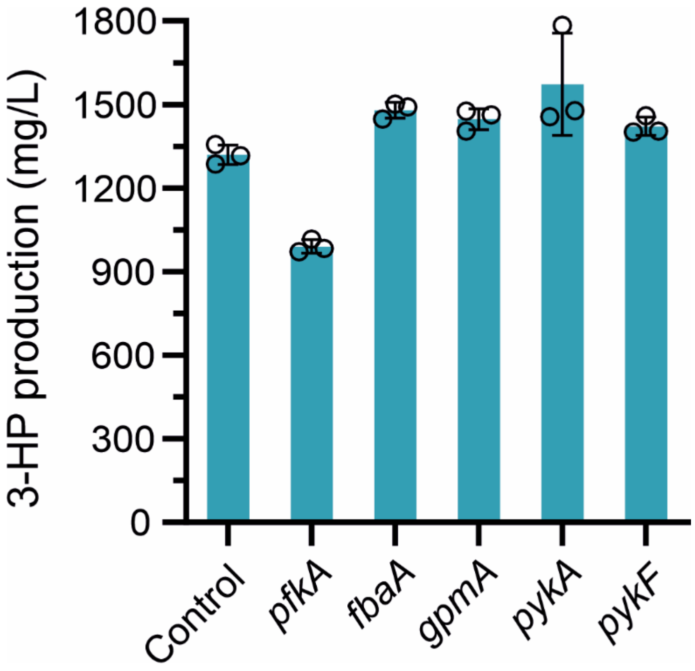

Supplementary Fig. 8 Expression of glycolytic genes from *E. coli*.

Genes from *E. coli* were driven by the *pALD4* promoter. Each gene was integrated into the yeast chromosome XII-1 locus in strain R30c, respectively. All strains were grown in a specific minimal medium with 2% xylose as the carbon source. All data presented in this figure represent the mean from n=3 biologically independent samples, with error bars indicating the standard deviation. Source data are provided as a Source Data file.

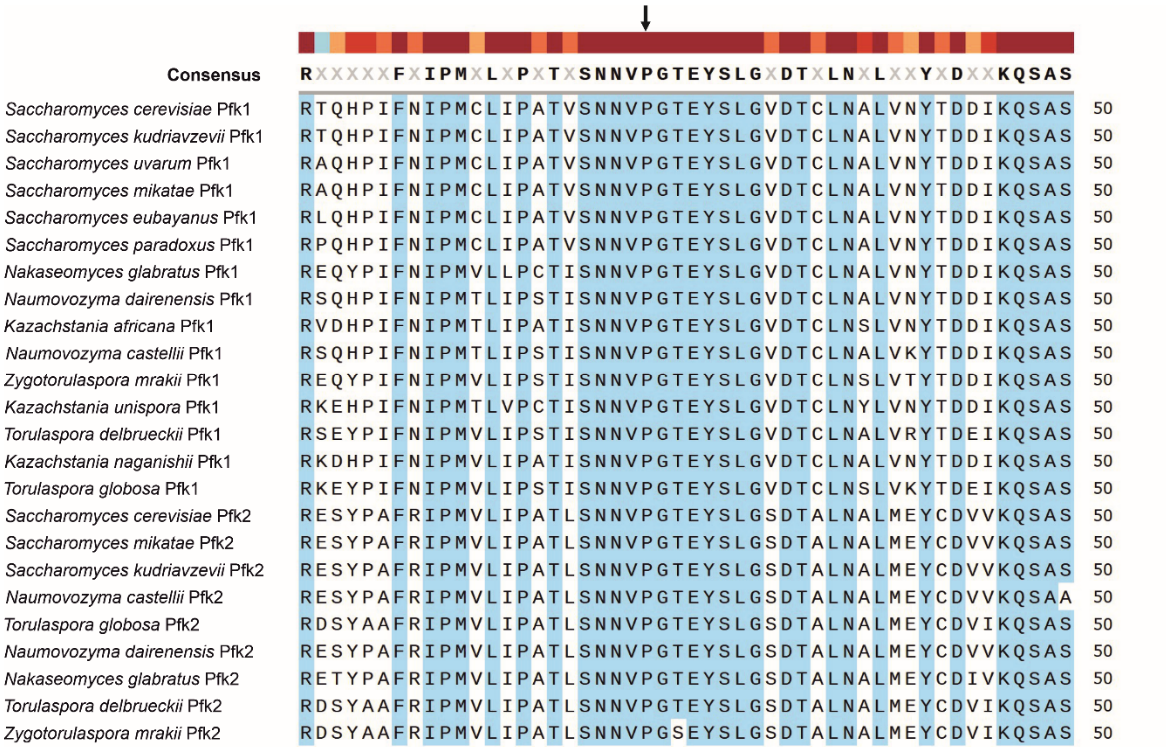

Supplementary Fig. 9 Multiple sequence alignments of Pfk1 and Pfk2 from different budding yeasts.

The BLAST search was conducted on the NCBI database, using the amino acid sequences of Pfk1 and Pfk2 as the query sequences. Sequences that exhibited greater than 80% homology to either Pfk1 or Pfk2 were selected for further analysis. These selected sequences were then aligned using the ClustalW algorithm. In the sequence alignment, regions matching the consensus sequence are highlighted in color. The colored bar above the alignment represents the degree of homology, with black arrows pointing to the sites of allosteric regulation by ATP.

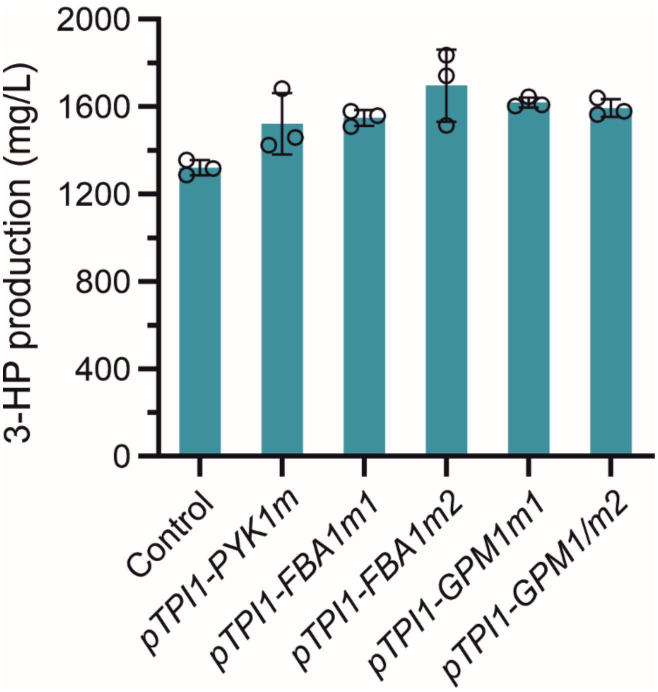

Supplementary Fig. 10 Overexpression of mutated glycolytic genes.

Genes were driven by the *pTPI1* promoter. Each gene was integrated into the yeast chromosome XII-1 locus in strain R30c, respectively. All strains were grown in a specific minimal medium with 2% xylose as the carbon source. All data presented in this figure represent the mean from n=3 biologically independent samples, with error bars indicating the standard deviation. Source data are provided as a Source Data file.

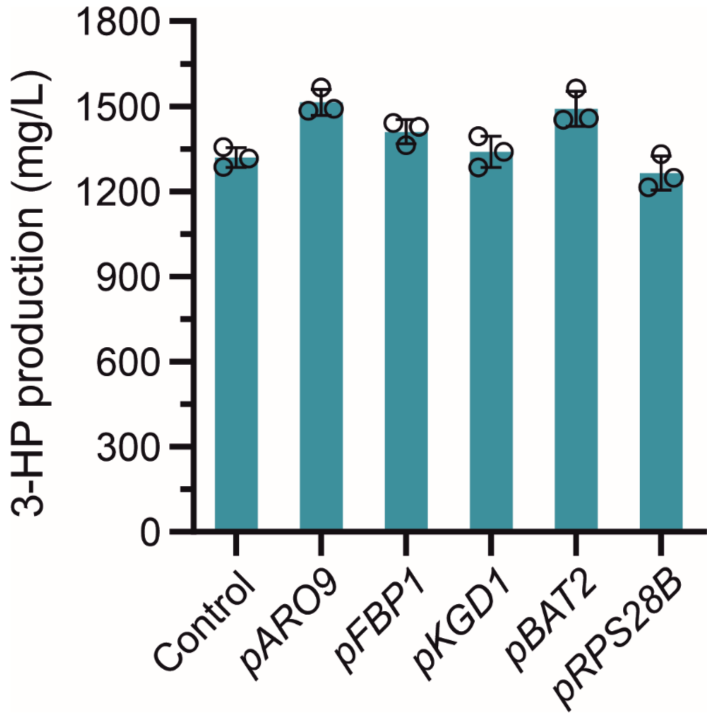

Supplementary Fig. 11 Fine-tuning the expression of Pfk27.

Pfk27 converts fructose-6-phosphate to fructose-2,6-bisphosphate, a potent allosteric activator of the glycolytic enzyme Pfk1/2. The expression of Pfk27 was regulated by five distinct promoters. Strain R30c was used as the control. All strains were grown in a specific minimal medium with 2% xylose as the carbon source. All data presented in this figure represent the mean from n=3 biologically independent samples, with error bars indicating the standard deviation. Source data are provided as a Source Data file.

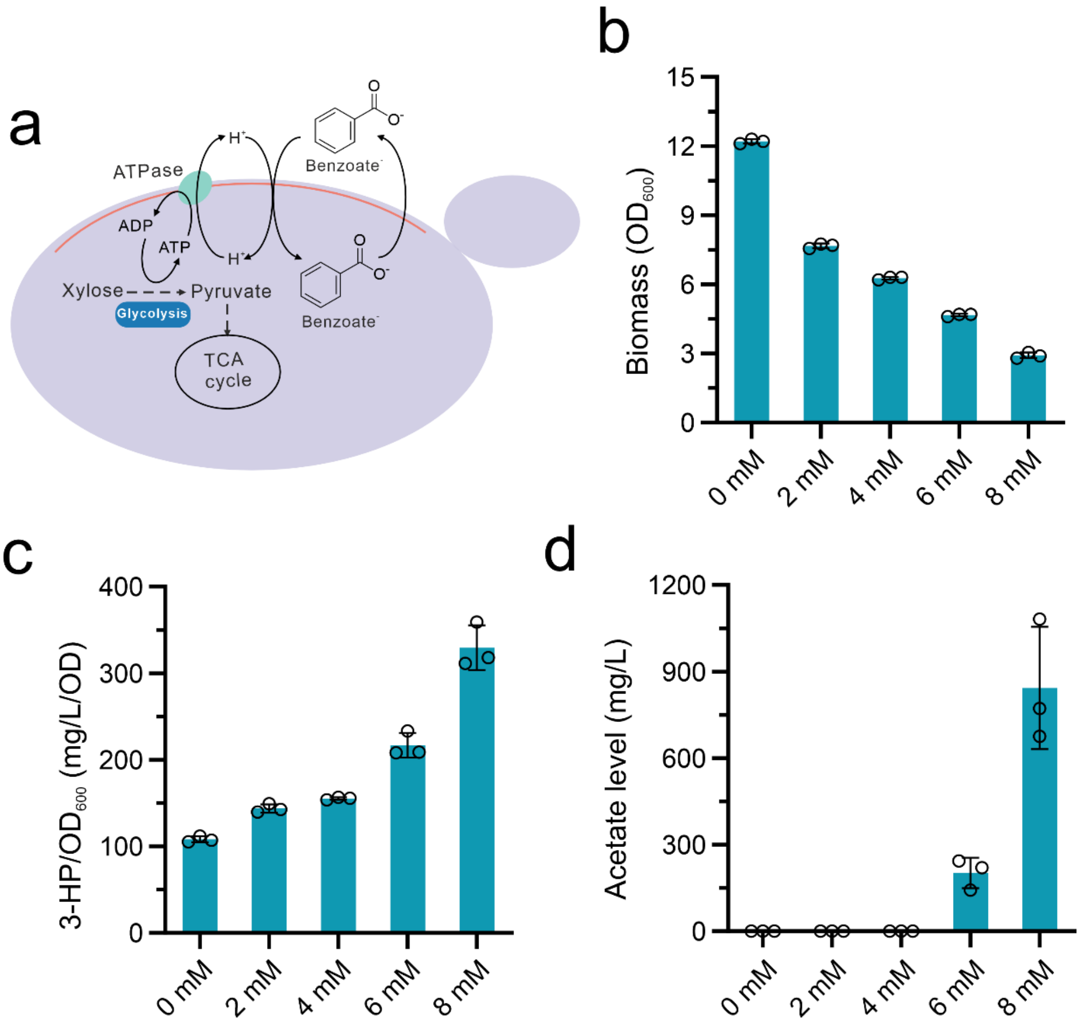

Supplementary Fig. 12 The effect of adding benzoic acid on yeast energy metabolism and 3-HP formation.

a, Schematic illustration of the link between benzoic acid transport and ATP consumption. A decrease in ATP further boosts glycolytic activity to generate more ATP. **b**, Biomass formation after adding different concentrations of benzoic acid. **c**, 3-HP production relative to OD cells in xylose media with various benzoic acid concentrations. **d**, Acetate production in the presence of various benzoic acid concentrations. All strains were grown in a specific minimal medium with 2% xylose containing different concentrations of benzoic acid. All data presented in this figure represent the mean from n=3 biologically independent samples, with error bars indicating the standard deviation. Source data are provided as a Source Data file.

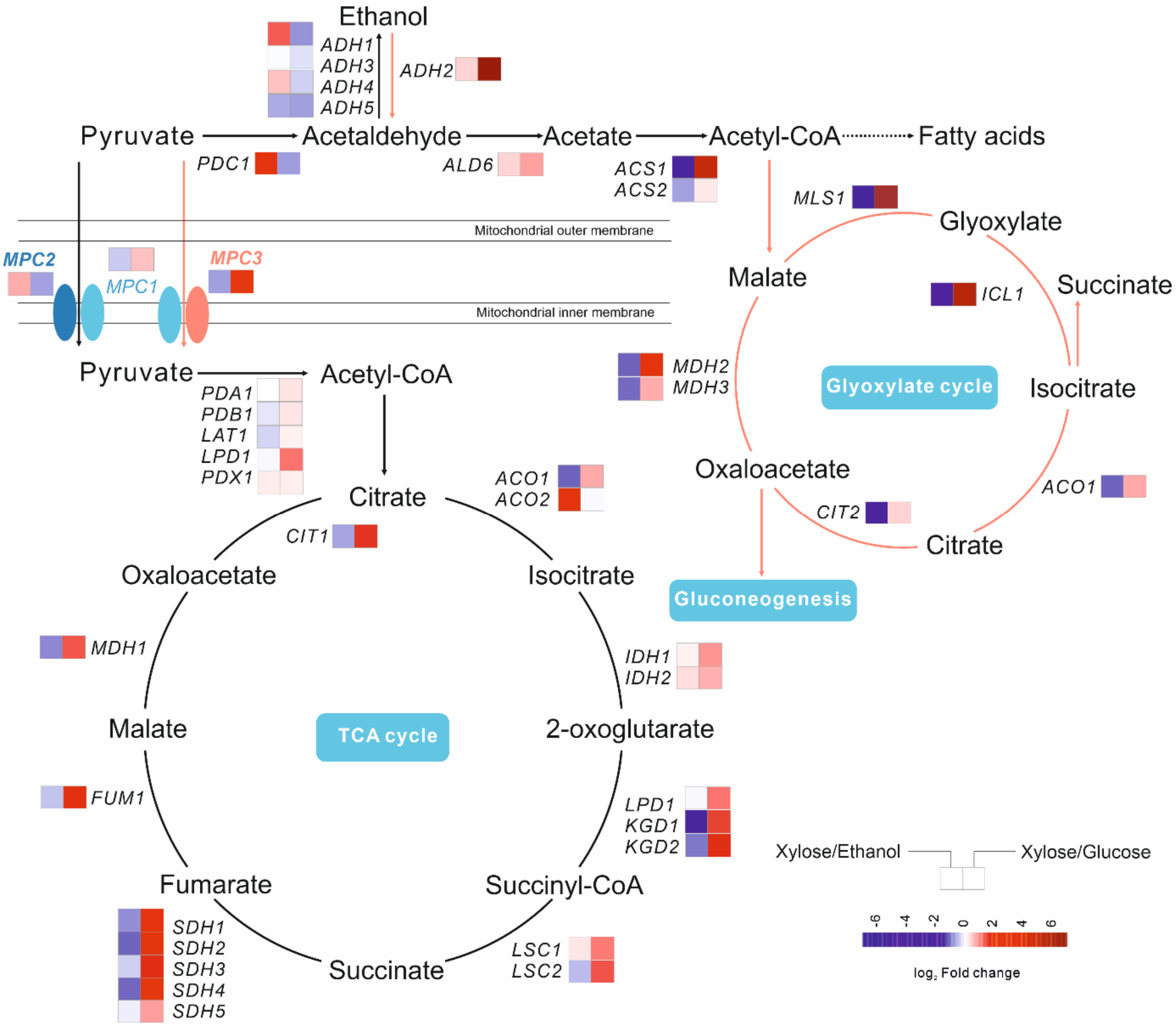

Supplementary Fig. 13 Transcriptional changes of genes involved in TCA cycle, glyoxylate cycle, and pyruvate bypass pathway.

The left squares represent transcriptional fold changes for strains grown on xylose versus ethanol, while the right squares represent transcriptional fold changes for strains grown on xylose versus glucose. Red arrows indicate the xylose metabolism reactions. Genes for each catalytic step are labeled adjacent to the arrows.

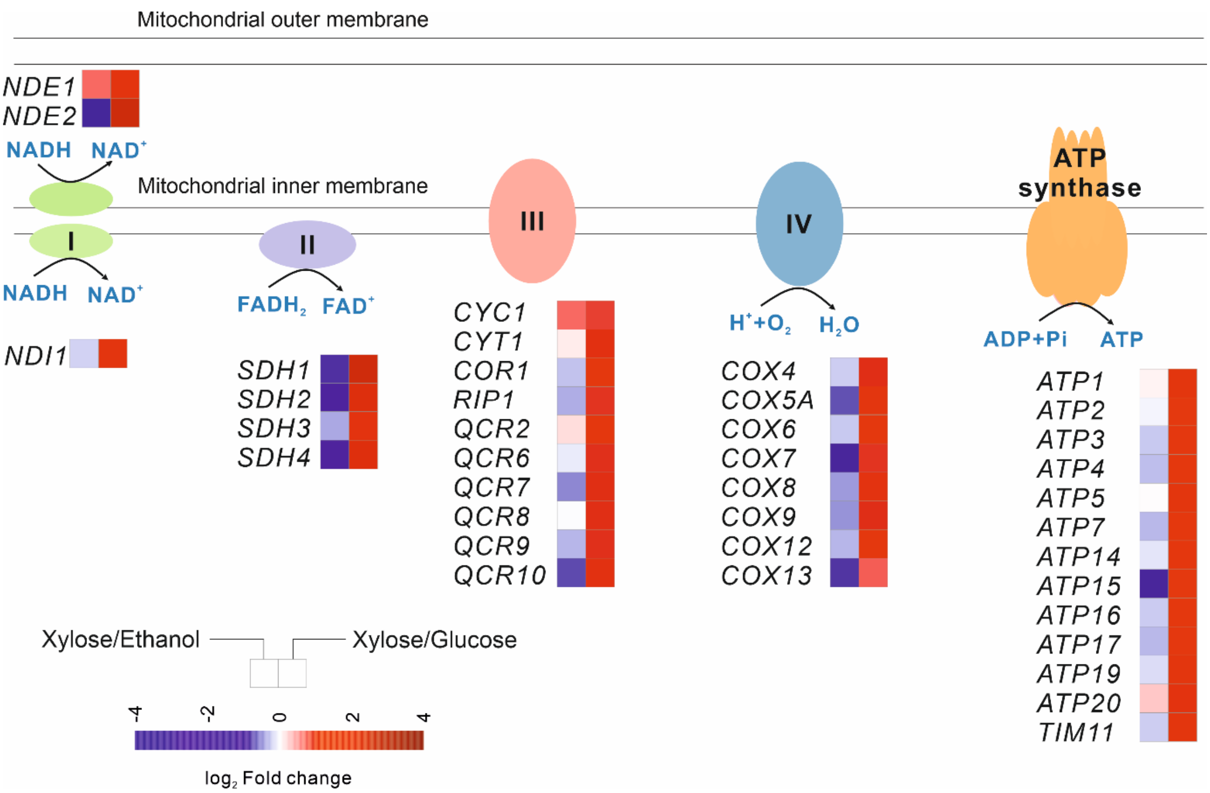

Supplementary Fig. 14 Transcriptional changes of genes involved in electron transport chain.

The left squares represent transcriptional fold changes for strains grown on xylose versus ethanol, while the right squares represent transcriptional fold changes for strains grown on xylose versus glucose. Genes for complex I-IV and ATP synthase are labeled below.

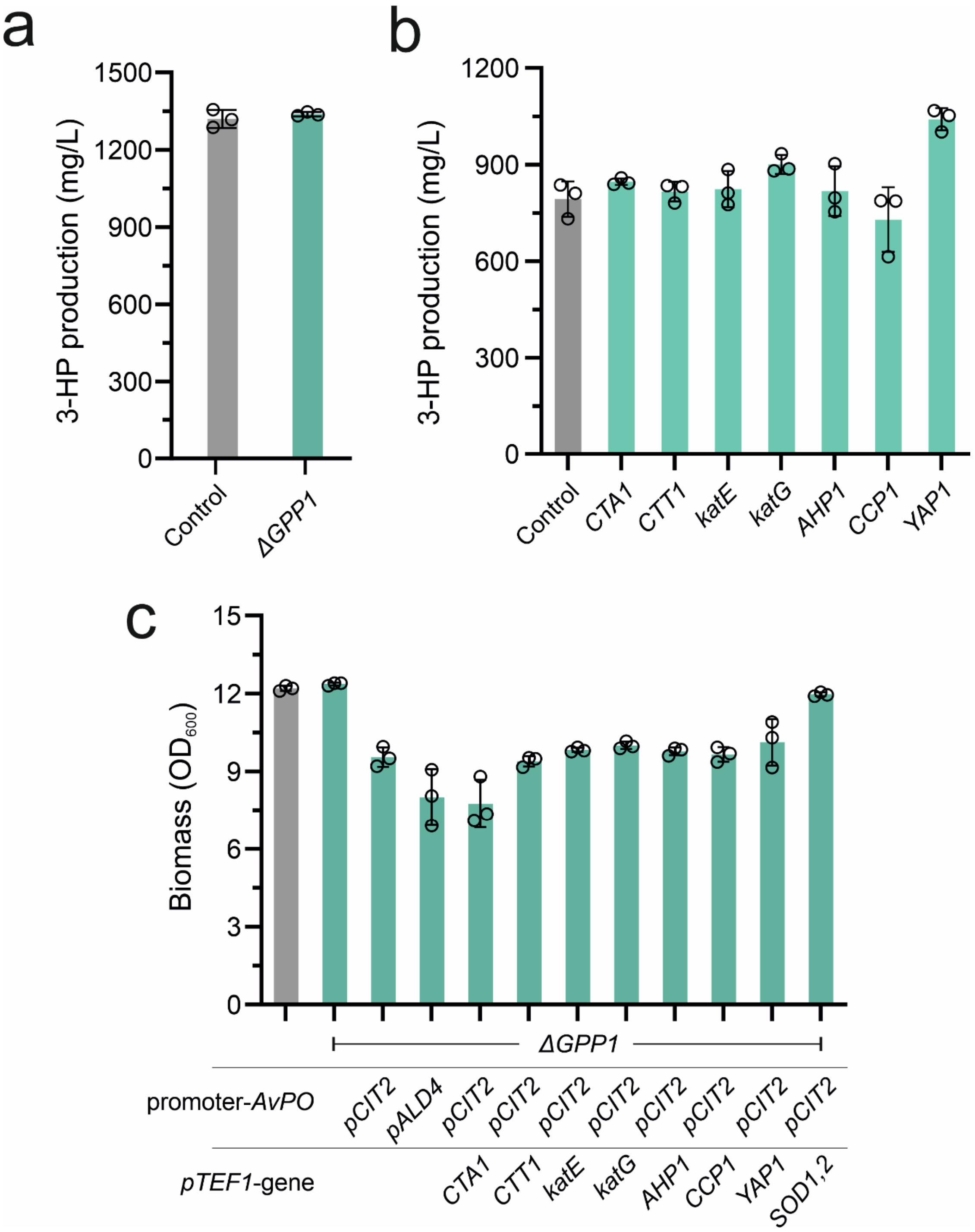

Supplementary Fig. 15 Evaluating the heterologous PO-PTA pathway.

a, Examination of the impact of *GPP1* deletion in strain R30c. **b-c**, Evaluation of various oxidative stress-responsive genes. Cta1 and Ctt1, yeast catalases; KatE and KatG, catalases from *E. coli*; Ahp1, thiol-specific peroxiredoxin; Ccp1, cytochrome-c peroxidase; Yap1, a transcription factor essential for oxidative stress tolerance; Sod1,2, superoxide dismutase. For (**a**), strain R30c was used as the control. For (**b**), strain R80p, where the *GPP1* gene was deleted and the genes involved in PO-PTA pathway were expressed, served as the control. For (**c**), strain R70, derived from strain R30c with *GPP1* deleted, was used as a control strain. All strains were cultivated in flasks containing a minimal medium with 2% xylose. All data represent the mean derived from n=3 biologically independent samples, with error bars indicating the standard deviation. Source data are provided as a Source Data file.

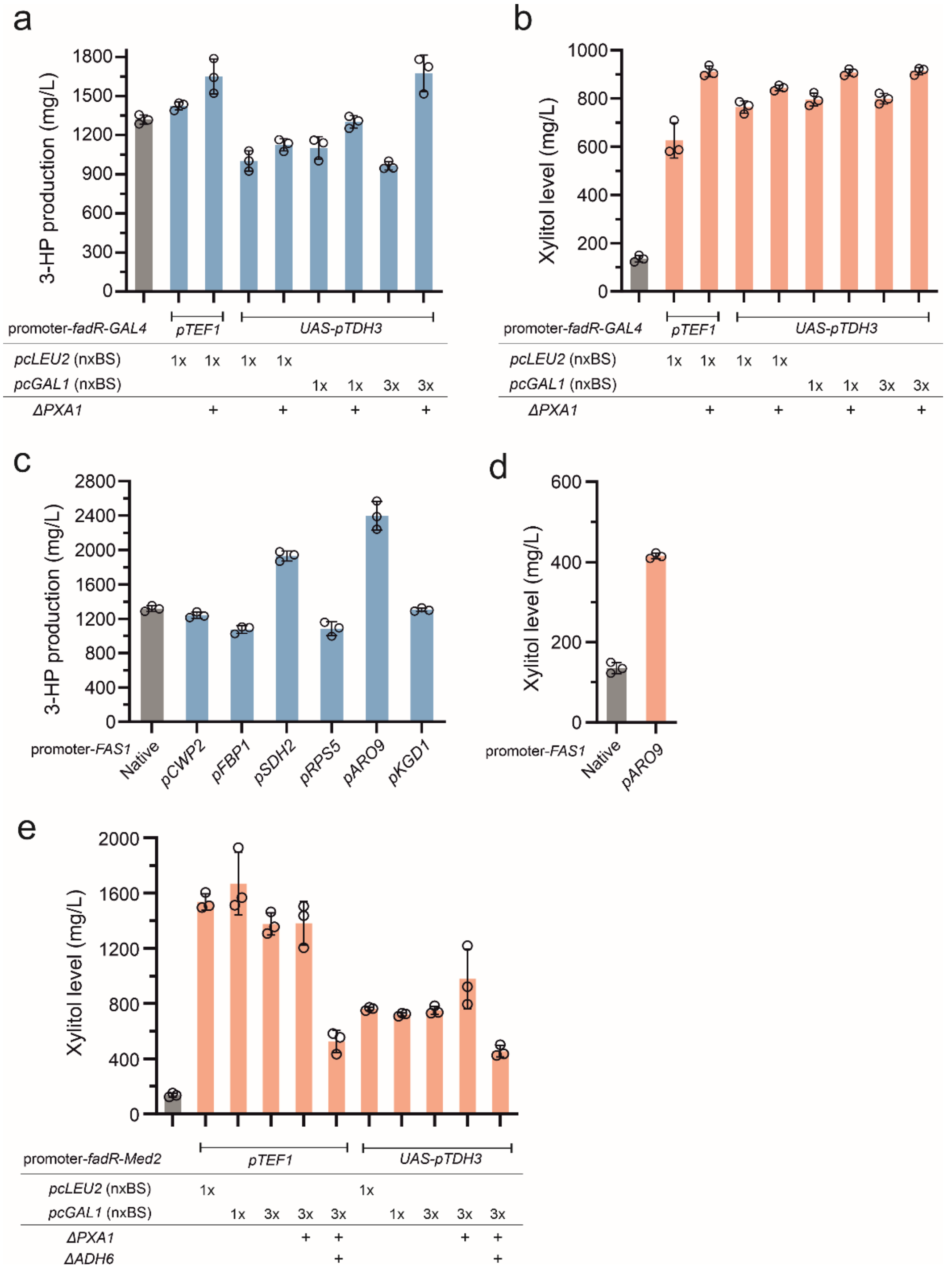

Supplementary Fig. 16 Decreasing metabolic flux towards fatty acid synthesis.

a, Regulating metabolic flux by monitoring intracellular acyl-CoA levels. FadR was fused with transcriptional activator Gal4. Either one or three FadR binding sites were integrated into the upstream regions of the *pLEU2* or *pGAL1* core promoters. **b**, Xylitol production in strains employing the biosensor constructed in (**a**). **c**, 3-HP production in strains with fine-tuned Fas1 expression. **d**, Xylitol production in strains with fine-tuned Fas1 expression. **e**, Xylitol production in strains containing the biosensor described in **Fig. 5h**. Strain R30c served as the control. All strains were grown in flasks containing a minimal medium with 2% xylose. All data represent the mean derived from 3 biologically independent samples, with error bars indicating the standard deviation. Source data are provided as a Source Data file.

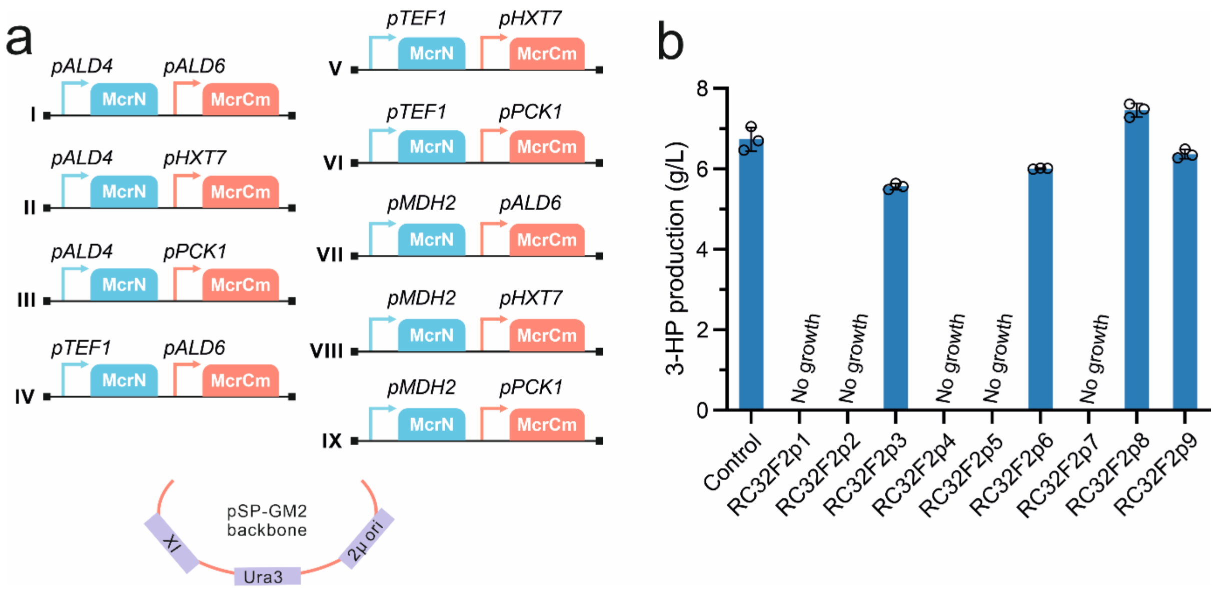

Supplementary Fig. 17 Optimizing the expression of *MCR* in plasmids in xylose medium.

a, Schematic illustration of plasmid constructions. McrN, driven by promoters *pALD4*, *pTEF1*, or *pMDH2*, and McrCm, driven by promoters *pALD6*, *pHXT7*, or *pPCK1*, were integrated into a high-copy plasmid containing XI. **b**, 3-HP production in strains harboring the constructed plasmids. Strain RC32F2p, as shown in **Fig. 6b**, served as the control. Strains with the corresponding plasmids were grown in flasks containing a minimal medium with 2% xylose. All data represent the mean derived from n=3 biologically independent samples, with error bars indicating the standard deviation. Source data are provided as a Source Data file.

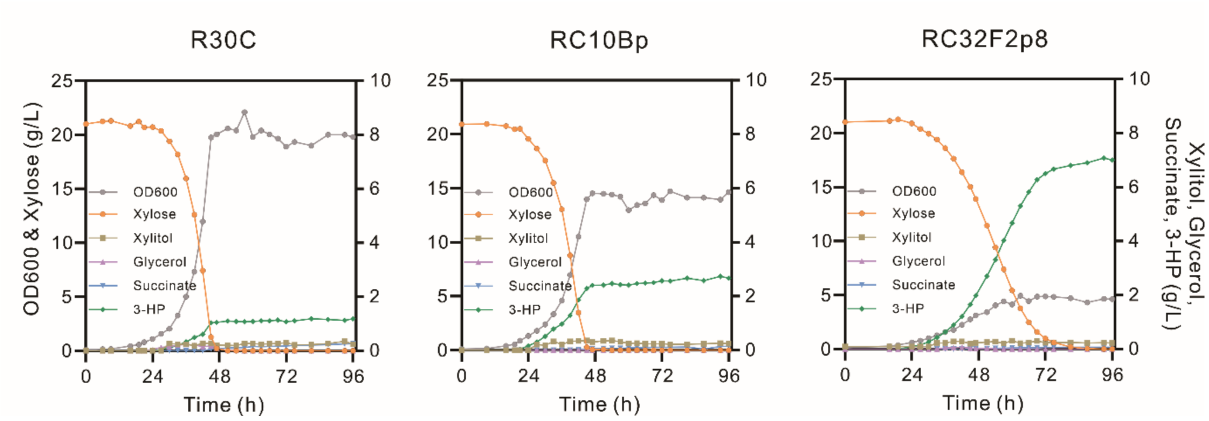

Supplementary Fig. 18 Fermentation of engineered strains.

The engineered low-level 3-HP producer, R30C (left), the medium-level 3-HP producer, RC10Bp (middle), and the high-level 3-HP producer, RC32F2p8 (right), were cultivated in a fermentor. Specifically, all strains were grown in a minimal medium supplemented with 2% xylose, at 30°C and pH 6.0. Source data are provided as a Source Data file.

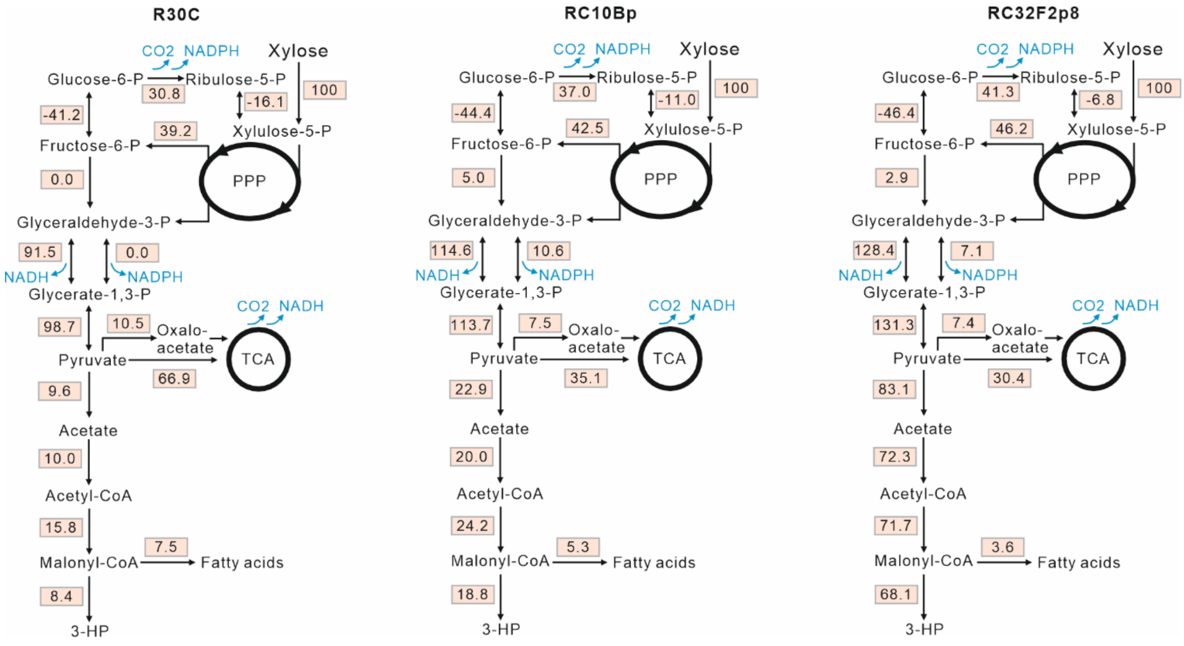

Supplementary Fig. 19 Calculated metabolic flux distributions via parsimonious flux balance analysis (pFBA).

Strains R30C (left), RC10Bp (middle), and RC32F2p8 (right) were used for pFBA. Based on pFBA, the fluxes of various metabolites were calculated relative to the uptake of 100 mmol of xylose. All strains were grown in fermentors containing a minimal medium supplemented with 2% xylose.

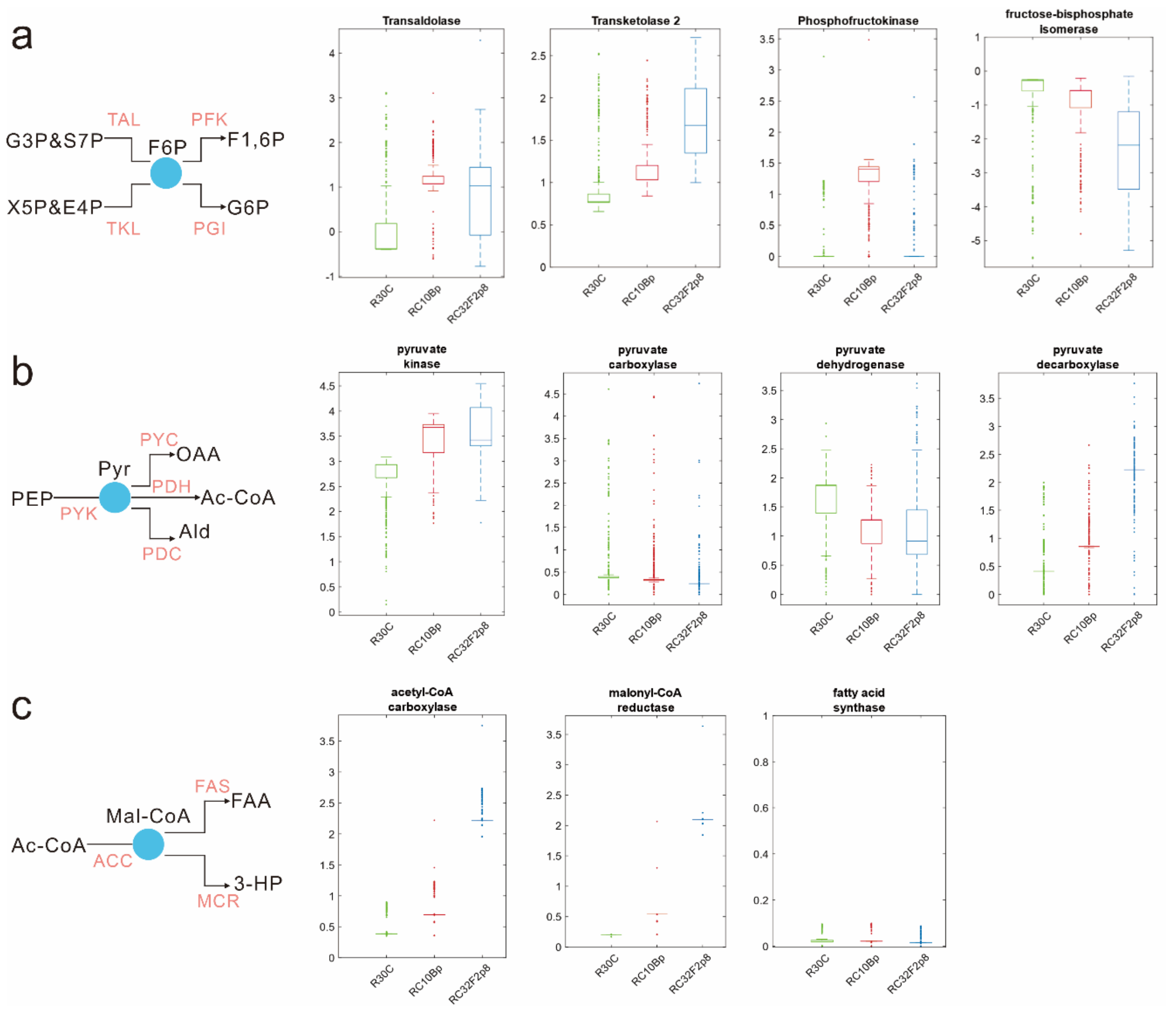

Supplementary Fig. 20 The distribution of calculated flux at three important nodes.

Figures **a-c** show the distribution of calculated flux at the F6P, Pyr, and Mal-CoA nodes, respectively. Each reaction's flow rate was randomly calculated 1000 times. The three strains were grown in fermentors containing a minimal medium supplemented with 2% xylose. G3P, Glyceraldehyde 3-phosphate; S7P, Sedoheptulose 7-phosphate; X5P, Xylulose 5-phosphate; E4P, Erythrose 4-phosphate; F6P, Fructose 6-phosphate; F1,6P, Fructose 1,6-bisphosphate; G6P, Glucose 6-phosphate; PEP, Phosphoenolpyruvate; Pyr, Pyruvate; OAA, Oxaloacetate; Ac-CoA, Acetyl-CoA; Mal-CoA, Malonyl-CoA; FAA, Fatty Acid. The horizontal line in the box represents the median of the data. The upper and lower edges of the box correspond to the 25th and 75th percentiles of the data, respectively.

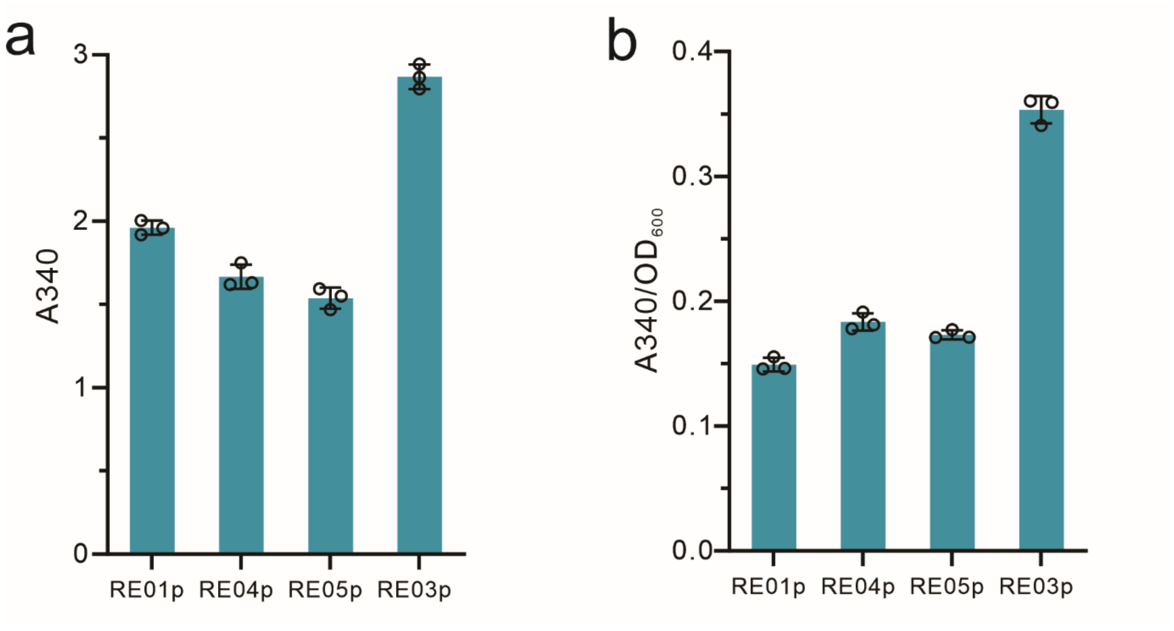

Supplementary Fig. 21 Assess the production of Flaviolin in the background strain for 3-HP synthesis.

a, The *MCR* genes were deleted in strains R30C, RC05p, RC10Bp, and RC32F2p8, and the *rppA* gene was introduced into these strains, resulting in the generation of strains RE01p, RE04p, RE05p, and RE03p, respectively. **b,** The production of flaviolin per OD of biomass. Strains were grown in flasks containing a minimal medium with 2% xylose. All data represent the mean derived from n=3 biologically independent samples, with error bars indicating the standard deviation. Source data are provided as a Source Data file.
