# Supplementary material for: Modular deregulation of central carbon metabolism for efficient xylose utilization in Saccharomyces cerevisiae
Source: Nat Commun. 2025 May 16;16:4551. doi: 10.1038/s41467-025-59966-x (PMC12084563; doi:10.1038/s41467-025-59966-x)
Supplement: Supplementary file 2 — Description of additional supplementary files [file 41467_2025_59966_MOESM2_ESM.docx]

File Name: Supplementary Data 1

Description: Plasmids used in this study. These plasmids include those for gene expression in yeast, protein mutation, and gRNA production.

File Name: Supplementary Data 2

Description: Strains used in this study. For each strain, we recorded its name, genotype, parental strain, and genetic modifications in the parental strain.

File Name: Supplementary Data 3

Description: Altered transcript levels in xylose and glucose media. Gene names, fold changes in transcript levels, and p-values are provided. The p-values were calculated using the Benjamini–Hochberg method.

File Name: Supplementary Data 4

Description: Promoters used in this study. The amplified sequence for each promoter region is provided.

File Name: Supplementary Data 5

Description: Codon-optimized genes in this study. All genes exogenously expressed in *Saccharomyces cerevisiae* were codon-optimized and synthesized.

File Name: Supplementary Data 6

Description: The scored transcription factors from the entire genomic catalog of yeast genes (input data 1). The p-values were calculated using the Benjamini–Hochberg method.

File Name: Supplementary Data 7

Description: The scored transcription factors from a subset of genes crucially linked to central metabolism (input data 2). This subset included genes related to glycolysis, gluconeogenesis, the tricarboxylic acid (TCA) cycle, the pentose phosphate pathway (PPP), oxidative phosphorylation, respiration metabolism, pyruvate metabolism, the glyoxylate cycle, as well as carbon transport and metabolism. The p-values were calculated using the Benjamini–Hochberg method.

File Name: Supplementary Data 8

Description: The performance of six different engineering strains. These strains were cultivated in a minimal medium in flasks containing 2% xylose as the sole carbon source.

File Name: Supplementary Data 9

Description: Codes used for the flux balance analysis (FBA). The codes used for constructing a new metabolism model from the parental strain IMX581 are provided in the file named baseModel. The codes for conducting parsimonious flux balance analysis (pFBA) in strains R30C, RC10Bp, and RC32F2p8 are located in the respective files R30C_analysis, RC10Bp_analysis, and RC32F2p8_analysis. The codes utilized for calculating random samplings of the solution space in strains R30C, RC10Bp, and RC32F2p8 are stored in the files randomSamplingR30C, randomSamplingRC10Bp, and randomSamplingRC32F2p8, respectively.
